# Autoimmune Disorders of the Nervous System: Pathophysiology, Clinical Features, and Therapy

**DOI:** 10.3389/fneur.2021.664664

**Published:** 2021-04-14

**Authors:** Satyakam Bhagavati

**Affiliations:** Department of Neurology, Downstate Medical Center, State University of New York College of Medicine, New York, NY, United States

**Keywords:** autoimmunity, nervous system, clinical, therapy, pathophysiology

## Abstract

Remarkable discoveries over the last two decades have elucidated the autoimmune basis of several, previously poorly understood, neurological disorders. Autoimmune disorders of the nervous system may affect any part of the nervous system, including the brain and spinal cord (central nervous system, CNS) and also the peripheral nerves, neuromuscular junction and skeletal muscle (peripheral nervous system, PNS). This comprehensive overview of this rapidly evolving field presents the factors which may trigger breakdown of self-tolerance and development of autoimmune disease in some individuals. Then the pathophysiological basis and clinical features of autoimmune diseases of the nervous system are outlined, with an emphasis on the features which are important to recognize for accurate clinical diagnosis. Finally the latest therapies for autoimmune CNS and PNS disorders and their mechanisms of action and the most promising research avenues for targeted immunotherapy are discussed.

## Introduction

The immune system has evolved to have the extraordinary ability to protect us from a vast multitude of infectious agents. However, in an estimated 4.5% of individuals the immune system attacks the very individual it is designed to protect (1). Abnormal immune responses against self can result in more than 80 autoimmune diseases (2) including about 30 autoimmune disorders of the nervous system.

In this article autoimmune disorders of the central and peripheral nervous system are discussed, including disorders where an auto-antigen has yet to be defined. These include central nervous system demyelinating disorders such as multiple sclerosis and neuromyelitis optica, paraneoplastic, and other autoimmune encephalomyelitis and autoimmune inflammatory myositis and demyelinating neuropathies. Neuroinflammatory disorders due to other causes such as cerebral degeneration or nervous system involvement secondary to systemic autoimmune disorders such as systemic lupus erythematosus are not included in this discussion.

### Failure of Self Tolerance and Development of Autoimmunity

What are the factors that lead to the breakdown of tolerance in some individuals?

### Genetic Susceptibility

In multiple sclerosis (MS), concordance in monozygotic twins of about 25% compared to ~5% in dizygotic twins (3); and in myasthenia gravis (MG) concordance of ~35% in monozygotic twins compared to ~5% in dizygotic twins (4), suggests the contribution of genetic factors to disease causation. Also higher incidence in females of most autoimmune diseases [~80% of all patients (5); neuro-myelitis optica, NMO, 9:1 (6); MS 3:1, early onset MG 3:1], points to the importance of still poorly understood X chromosome factors in genetic susceptibility (7), although effects of sex hormones on immune responses may also play an important role (8).

The MHC locus makes a greater contribution to risk of autoimmune disease than any other loci. MHCII molecules on antigen presenting cells capture peptides and present them to T cells. The MHC locus is also the most polymorphic in the human genome, with a differential ability to bind and efficiently present peptides. Such variations play an important role in the differences between individuals in their immune response to auto-antigens, and is the basis for the strong association between specific MHC alleles and predisposition to autoimmune disease (as in myasthenia gravis or multiple sclerosis). For example, the HLA-peptide T cell receptor binding geometry may play a crucial role in disease causation (9). An off-center binding topology (as has been demonstrated with a T cell clone isolated from a MS patient), may permit autoreactive T cell clones to escape negative thymic selection but still cause disease (10). In other examples of strong genetic predisposition, anti-LGI1 encephalitis shows a strong HLA association with the DRB1^*^07:01 allele, which is carried by 90% of patients vs. 13% of healthy controls (11); anti-IgLON5 disease is tightly associated with the DRB1^*^10.01 allele (12); CASPR2-antibody patients show over representation of HLA-DRB1^*^11:01 (13); MUSK-Myasthenia gravis is tightly associated with the HLADR14-DQ5 haplotype (14) and early onset anti-AChR MG with HLA-B^*^8:01 and late onset MG with HLA DRB1^*^ 15:01 (15).

In addition, genome wide association studies (GWAS) have identified hundreds of single nucleotide polymorphisms (SNP; variations in the genome at the single nucleotide level) which increase the risk of developing autoimmune disease by small to moderate amounts. Many of these SNPs are in non-coding regions of the genome and the molecular and cellular consequences of these subtle variations are complex and largely undefined. But it is known that autoimmune disease risk SNPs cluster in genes in key immunological pathways. A few examples of how inherited genetic variations can predispose individuals to autoimmune disease are: (1) A recent whole-genome sequence study showed that deletion of complement component C4A gene in the MHC class III region heightens risk for NMO and also SLE (16); (2) Interleukin-2 receptor (IL2R) signaling is essential for optimal stability and function of immunosuppressive T_reg_ cells. Polymorphisms in genes in this pathway such as the IL2R locus leads to decreased responsiveness to IL2 and increased susceptibility to MS (17); (3) Nucleotide variations in the promoter of the muscle acetyl choline receptor (AChR) gene, associated with early onset of myasthenia gravis, prevent binding of interferon 8 which leads to decreased expression of AChR gene in thymic medullary cells (18). This may raise the threshold and impair clonal deletion of AChR auto-reactive T cells in the thymus; (4) Rare polymorphisms in coding sequences of genes important in immunity (for example in perforin, important in granzyme mediated lymphocyte cytotoxicity) increase MS risk (19).

### Factors Which May Trigger Autoimmune Disease

#### Infections

Although not fully understood, epidemiological and clinical evidence suggests that autoimmune disease may be triggered by infections (20). In MS, it has been proposed that lifelong infection with EB virus or CMV infection creates a pool of auto-reactive memory T cells which may be reactivated when exposed to antigens released after CNS injury (21). Similarly high rates of preceding infection have been noted before MOG- antibody associated disease. Molecular mimicry between host self-antigens and microbial antigens is a leading hypothesis to explain this association (22). Low avidity, circulating auto-reactive T cells which may have unusual binding conformations with self-MHC and do not cause pathology, may be activated by cross-reaction with high affinity or high abundance pathogenic peptides derived from infectious agents. Cross reactivity is plausible as a single T cell receptor can recognize a broad range of different epitopes (TCR degeneracy) (23, 24) and importantly, because the peripheral T cell activation threshold is lower than that for negative selection in the thymus (25). Classically, molecular mimicry was postulated on the basis of shared epitopes between Group A streptococci and cardiac glycoproteins leading to rheumatic fever. In the nervous system a strong case has been made for the development of Guillain Barre syndrome (GBS) after Campylobacter jejuni infection. Antibodies against *C. jejuni* cell wall lipo-oligosaccharide antigens cross-react against gangliosides (nearly identical self-antigens), which are major constituents of the nerve cell membrane. An animal model in rabbits in which immunization with *C. jejuni* lipo-oligosaccharide results in a neuropathy similar to GBS provides support for this hypothesis (26). It is important to note, however, that <0.1% of *C. jejuni* infected individuals develop cross-reactive antibodies and Guillain Barre syndrome (27), raising questions about the broader relevance of the molecular mimicry model for autoimmune disease causation.

Alternatively, primary CNS tissue damage (after Herpes simplex encephalitis, for example, which has been known to trigger NMDA encephalitis) (28), may lead to the release and export of self- antigens that drain into the CNS draining deep cervical nodes. The discovery of lymphatics in the dura mater draining to cervical lymph nodes has pointed to a path by which CNS antigens may reach peripheral immune tissue (29). In support of this, in experimental autoimmune encephalomyelitis (EAE), myelin antigens associated with dendritic cells have been found in the cervical lymph nodes of non-human primates (30). In this model, on presentation of CNS antigens to quiescent, auto-reactive lymphocytes in CNS draining lymph nodes, T or B cells could be activated. Activated CD4^+^T cells could then provide help to activate naïve B cells and also produce high amounts of cytokines like interferon λ and granulocyte macrophage colony stimulating factor (GM-CSF), which promote local inflammation and loss of blood-brain barrier integrity. Activated memory B cells migrating back to the CNS could undergo antigen driven affinity maturation (possibly in ectopic tertiary lymphoid structures in the meninges as has been described in MS and NMOSD) and differentiate into antibody producing plasma cells, explaining the observed intrathecal synthesis of autoantibodies.

In addition accumulation of somatic point mutations in immunoglobulin variable (V) gene of B cells proliferating in response to pathogens, provides the basis for extraordinary antibody diversity and affinity maturation in response to foreign antigens. A genealogical analysis of antibodies secreted by antigen specific B cell clones from autoimmune disease patients has shown that unmutated common ancestors of auto-antibodies (for example to the autoantigen desmoglein3 in pemphigus) (31) or to nuclear autoantigens in SLE (32) do not bind to the respective self-antigen. Thus, somatic mutations in immunoglobulin (V) genes in B cell clones, proliferating in response to infection, while generating a massive diversity of antibodies, may randomly and inadvertently generate antibodies that occasionally bind and cross-react with self-antigens, even in the absence of structural molecular mimicry with pathogenic antigens (33). This may be another mechanism for breach of self-tolerance.

Another mechanism to consider is secondary to the presence of natural autoantibodies, found in individuals even without prior antigenic experience. The sera of healthy individuals contain an enormous number of such antibodies that are poly-reactive and bind with low affinity with various self and non-self-antigens. By chronically reacting with self-antigens autoreactive B cells achieve non-responsiveness to them (anergy) but, it has been speculated that they may be susceptible to activation by infection or tissue damage leading to the production of high affinity antibodies and autoimmune disease (34, 35).

#### Microbiota

The microbiota is an ecosystem of trillions of microrganisms that reside on mucosal surfaces and skin in beneficial co-existence with the host. Recently the surprising connection between disordered intestinal microbiota and brain autoimmunity has been made (36). This was based on the finding that germ free mice were resistant to developing EAE (the classic animal model for multiple sclerosis), but susceptibility to EAE was restored in mice exposed to commensal bacteria (37). Also, fecal transplants from MS patients into germ free mice have been shown to result in a higher incidence or severity of EAE (38, 39). Another recent study of the antigen reactivity of a CD4^+^ T cell clone isolated from brain tissue of a multiple sclerosis patient showed reactivity to an ubiquitous auto-antigen guanosine diphosphate (GDP)–L- fucose synthetase, an evolutionarily conserved enzyme also used by gut microbiota (40). Forty percent of T cell clones isolated from CSF of MS patients reacted to this antigen and, notably, also to myelin. Thus, in this hypothesis, CD4^+^ T cells could be activated in the gut against microbial gut GDP-L-fucose synthetase, travel to the CNS and cross-react with myelin or other CNS antigens resulting in disease (41). Similarly, in another study of a mouse model of autoimmune uveitis, it was shown that non-cognate antigen from commensal intestinal microbes can activate eye specific auto-reactive T cells resulting in autoimmune uveitis (42).

#### Epitope Spreading

An alternative explanation for the finding described above could be that after the initial CNS inflammation induced by a specific CNS autoantigen, CNS injury results in the release of cryptic antigens which provoke a secondary CD4^+^ T cell response. In a similar manner, CSF immunoglobulin oligoclonal bands, produced by clonally expanded CSF B cells suggests intra-thecal antibody synthesis and is a hallmark of multiple sclerosis. The CNS antigenic target of these antibodies has been long sought to elucidate MS pathogenesis. However, a recent study based on aligning patient specific CSF oligoclonal peptides to patient specific immunoglobulin transcriptome derived from CSF B cells, showed that the B cell response in MS is heterogenous and non-specific and directed against ubiquitous intracellular auto-antigens (43) released during tissue destruction, implying unmasking of cryptic epitopes and cascading inflammatory damage.

#### Tumors

Ectopic expression of neural proteins (onconeural antigens) in cancer can trigger an aberrant autoimmune response misdirected against the nervous system (para-neoplastic disorders, see Table 1). For example in ovarian teratoma (associated with NMDA encephalitis), the tumor itself contains mature or immature neural tissue (52). Antigens released by dying tumor cells may be taken up, processed and presented by antigen presenting cells in regional lymph nodes to activate T cells. Furthermore, ectopic germinal centers have been described within ovarian teratomas in some patients who present with NMDAR encephalitis (53). These could be a source of continued auto-antibody production.

**Table 1:** Main features of autoimmune diseases of the central nervous system.

**Target antigen**	**Auto-Ab pathogenic**	**Auto-Ab marker**	**Main auto-antibody effect/pathology**	**Main clinical features**	**Tumor association (%)**	**Tumor types (%)**
**Pathogenic autoantibodies against neuronal cell surface and synaptic proteins; Predominantly B cell (Antibody mediated); Generally responsive to immunotherapy**						
Glu N1	NMDAR IgG1		Autoantibodies cause reduction of cell surface NMDAR secondary to cross linking, internalization. Disruption of NMDAR-EPHB2 interaction leads to dispersal and loss of NMDAR from synaptic sites and disrupted glutamatergic transmission; reduced LTP; increased cortical excitability. Brain shows little neuronal loss; no complement activation; mainly CD4^+^ T cells, B cells. Markers of cytotoxicity (granzyme–B, perforin) scant.	Median age 22, 80% women, Hallucinations, delusions, memory loss, mania, catatonia, seizures, confusion, coma Oro-facial and limb dyskinesias, choreo-athetosis, dystonic postures, autonomic dysfunction: tachycardia, hypertension, hyperthermia	~60	Ovarian teratoma
LGI1 Secreted Synaptic protein	LGI1 IgG4		Autoantibodies cause reversible reduction of synaptic AMPAR and Kv1.1 by disrupting interaction of LGI1 with pre and post synaptic proteins ADAM23 and ADAM22; increased epileptiform activity in hippocampal CA3 neurons secondary to Kv1.1 inactivation. Neuronal cell loss; immunoglobulin and complement deposition.	Median age 60, M:F, 2:1 Limbic encephalitis; Facio-brachial or crural dystonic seizures, brief (<3 s), repetitive (median 50 per day) Hyponatremia	5–10%	Thymoma
GluA1 GluA2	AMPAR		Autoantibodies cause cross linking, internalization and decrease of synaptic AMPAR. Mild perivascular and interstitial lymphocytic infiltration in hippocampus.	Limbic encephalitis Non-focal enecephalitis	>50%	NSCLC ~35 Thymus ~30 Breast ~20
β1 subunit GABA_B_R	GABA_B_R		Directly blocks function of GABA_B_R without decreasing levels of cell surface receptor.	Limbic encephalitis Cerebellar Ataxia Opsoclonus-myoclonus	>50%	SCLC
GABA_A_R α1,β3 subunit	GABA_A_R		Crosslinking, internalization and downregulation of synaptic GABA_A_R	Intractable seizures, status epilepticus Altered behavior, cognition Dyskinesias	~30%	Thymoma
GlyRα1 subunit	GlyR		Autoantibodies cause cross linking, internalization of inhibitory glycine receptors (strychnine sensitive chloride channels) expressed mainly in brain stem, spinal cord and hippocampus.	Progressive encephalitis with rigidity and myoclonus (PERM); muscle stiffness, painful spasms, hyperkplexia, Brain stem encephalitis	—	
CASPR2	CASPR2 IgG4, IgG1		CASPR2 is an adhesion protein which promotes juxtaparanodal clustering of Kv1 channels in CNS and PNS. Antibodies react with juxtaparanodal region of myelinated peripheral nerves and also hippocampal inhibitory neurons.	Limbic encephalitis, insomnia, Neuromyotonia, neuropathic pain, dysautonomia, Morvan syndrome (above) Episodic ataxia	20%	Thymoma
mGluR1	mGluR1		Autoantibodies react with cerebellar Purkinje neurons and reduces Purkinje cell activity. Brain shows significant loss of Purkinje neurons in cerebellum.	Cerebellar ataxia Loss of taste	~10%	Hodgkin's lymphoma
mGluR5	mGluR5		Autoantibodies target mGluR5 which regulates rapid synaptic transmission in the hippocampus.	Ophelia syndrome: Confusion, agitation, memory loss, psychosis, seizures	<10%	Lymphoma
IgLON5 Cell adhesion molecule	IGLON5 IgG4		Autoantibodies cause a decrease of cell surface IgLON5. Neuronal loss, gliosis without inflammation; Neuronal hyperphosphorylated tau predominantly in hypothalamus, brain stem tegmentum, upper spinal cord.	Disturbed sleep, obstructive sleep apnoea, parasomnias, stridor, gait instability, chorea, supranuclear gaze palsy		
DPPX Extracellular subunit of Kv4.2 channel	DPPX IgG4;IgG1		Autoantibodies react with neuronal DPPX in hippocampus, cerebellum and myenteric plexus.	Prodromal diarrhea, weight loss, encephalitis, seizures, cerebellar ataxia, PERM	<10%	Lymphoma
**Non-pathogenic autoantibodies targeting intra-cellular antigens (classical paraneoplastic disorders); Predominantly cytotoxic CD8**^**+**^ **T cell mediated; Generally resistant to immunotherapy**						
Ma1, Ma2		Ma, Ma2	Ma1, Ma2 expressed in subcellular organelles including nucleoli of neurons. Neuronal loss, granzyme B +ve cytotoxic CD8+ cells.	Limbic, brain stem/diencephalic encephalitis, opthalmoplegia, excessive daytime sleepiness	>90%	Testicular ~50% NSCLC ~15% GI, Breast, Ovary, Colon
HuD (Elav4)		Hu (ANNA1)	Neuronal loss, gliosis, CD4+ and CD8+ cytotoxic T cell infiltration.	Sensory neuronopathy, limbic encephalitis, PCD, encephalomyelitis, gastroparesis, pseudo-obstruction, cardiac dysrhythmias	>90%	SCLC ~75% NSCLC ~10% Prostate, Breast, GI
CDR2, CDR2L		Yo (PCA1)	Multifocal inflammation in cerebellum, brain stem, spinal cord. Neuronal loss, mainly CD8+ T cell infiltration	PCD: Cerebellar ataxia, dysarthria, nystagmus	>90%	Ovarian ~60% Breast ~25% Fallopian tube
DNER		Tr	DNER is expressed by cerebellar Purkinje cells	PCD	>90%	Hodgkin's lymphoma
SOX1		SOX1		Eaton-Lambert Syndrome (see Table 3) PCD	>95%	SCLC~ 90%
NOVA1 NOVA2		Ri (ANNA2)	Neuronal loss in brain stem, cerebellum, spinal cord. Predominantly CD8+ T cell inflammatory infiltrates	~80% female; PCD: Cerebellar ataxia; Brain stem encephalitis, opsoclonus/myoclonus, laryngospasm, trismus	>85%	Breast ~50% Lung ~30%
Amphiphysin		Amphiphysin	Pre-synaptic protein important in clathrin mediated endocytosis which may cause decreased GABA/glycine uptake into vesicles and release	Encephalitis, stiff person syndrome, myelopathy, neuronopathy/neuropathy	>80%	SCLC ~60% Breast ~35%
CRMP5		CRMP5 (CV2)	Nerve fiber and myelin loss in brain, optic nerve, spinal cord, sensory ganglia, peripheral nerves. Mainly CD8+ T cell infiltrates	Encephalomyelitis; sensory, sensorimotor and autonomic neuropathy, chorea, optic neuritis, GI motility disorders	>90%	SCLC ~80% NSCLC ~5% Thymoma ~8%
MAP1B		MAP1B (PCA2)		Encephalomyelitis, ataxia, sensorimotor neuropathy	>90%	SCLC ~45% NSCLC ~25%
Kelch-like protein 11		Anti-Kelch11	Kelch 11 is a member of E3 ubiquitin ligase complex located intra-cellularly. T cell predominant inflammation in brain lesions and non-necrotizing granulomas.	Rhombencephalitis presenting with ataxia, vertigo, diplopia, hearing loss, seizures	~80%	Testicular germ cell tumors
Neurexin-3α		Anti-neurexin3α	Antibody decreases density of surface neurexin- 3α and total number of synapses in neurons undergoing development	Encephalitis Confusion, seizures	None	
D2R		Anti-D2R	Receptor internalization and decrease in D2R surface density	Basal ganglia encephalitis Parkinsonism, dystonia, psychiatric symptoms	None	
GAD65		Anti-GAD65	Clinical pathology only associated with high titer of antibodies. Likely cytotoxic T cell mediated pathology	Stiff person spectrum disorder; Cerebellar ataxia; epilepsy; limbic encephalitis	4%	Breast, lung, thyroid, thymoma

*AMPAR, αamino-3hydroxy-5-methyl-4-isoxazolepropionic acid receptor; ANNA, anti-neuronal nuclear antibody; CASPR2, contactin associated protein2; CRMP5, collapsing response mediator protein; DNER, delta and notch like epidermal growth factor-related receptor; DPPX, dipeptidyl aminopeptidase-like protein 6; GABA_A_R, γ-aminobutyric acid type A receptor; GABA_B_R, GABA type B receptor; GAD65, Glutamic acid decarboxylase 65; GluN1, glutamate receptor subunit of NMDA 1; GluA1/A2, glutamate receptor subunits of AMPAR; GlyR, Glycine receptor; IgLON5, Immunoglobulin like cell adhesion molecule 5; IgG4, immunoglobulin G4; Kv1.1, voltage gated potassium channel subunit; LGI1, leucine-rich glioma inactivated protein1; LTP, Long-term potentiation; MAPB1, microtubule associated protein 1B; mGluR, metabotropic glutamate receptor; NMDAR, N-methyl-D-aspartate receptor; NOVA, neuro-oncological ventral antigen; NSCLC, non-small cell lung cancer; PCD, paraneoplastic cerebellar degeneration; PERM, Progressive encephalomyelitis with rigidity and myoclonus; SCLC, small cell lung cancer. CSF complements serum and should also be tested in evaluation for auto-antibodies*.

*Rasmussen encephalitis which presents in children with focal seizures (epilepsia partialis continua) and encephalopathy is considered likely to be autoimmune in origin (44–51)*.

It is important to note that even though onconeural antigens occur frequently, only a small minority of cancer patients (<1%) develop paraneoplastic syndromes (54). This may be because, for auto-reactive T cells to be activated, mutations may have to be present in tumor antigens (55) (to be seen by the immune system as “foreign” antigens) and secondly, effective antigen presentation of the tumor peptide to T cells also requires compatibility with the patient's MHC molecules.

#### Vitamin D

Apart from its role in calcium homeostasis Vitamin D plays an important immunomodulatory, anti-inflammatory and anti-oxidant role (56). Several studies suggest that Vitamin D has an effect on the immune system by inhibiting B cell proliferation and antibody production and also by reducing T cell proliferation, Th1 and Th17 differentiation, increasing Treg cells and decreasing the secretion of pro-inflammatory cytokines (57).

Vitamin D is mainly produced in the skin after exposure to ultraviolet light and hypovitaminosis D has been linked to reduced exposure to sunlight in northern latitudes, where MS is more common, sparking interest in studying Vitamin D levels in MS. However, these studies have produce conflicting results. Vitamin D levels have been reported to correlate with decreased MS incidence (58) and lower levels have also been reported to increase susceptibility to the development of MS in pediatric patients (59). Other studies have, however, shown no difference in the level of Vitamin D between patients and controls (60) and a Cochrane review concluded no benefit from Vitamin D administration in reducing relapses, disability or MRI lesions in MS patients (61). The role of Vitamin D in nervous system autoimmune diseases is a subject of ongoing investigation.

## Clinical Features and Scientific Basis of Different CNS and PNS Autoimmune Diseases

**Figure 1:**
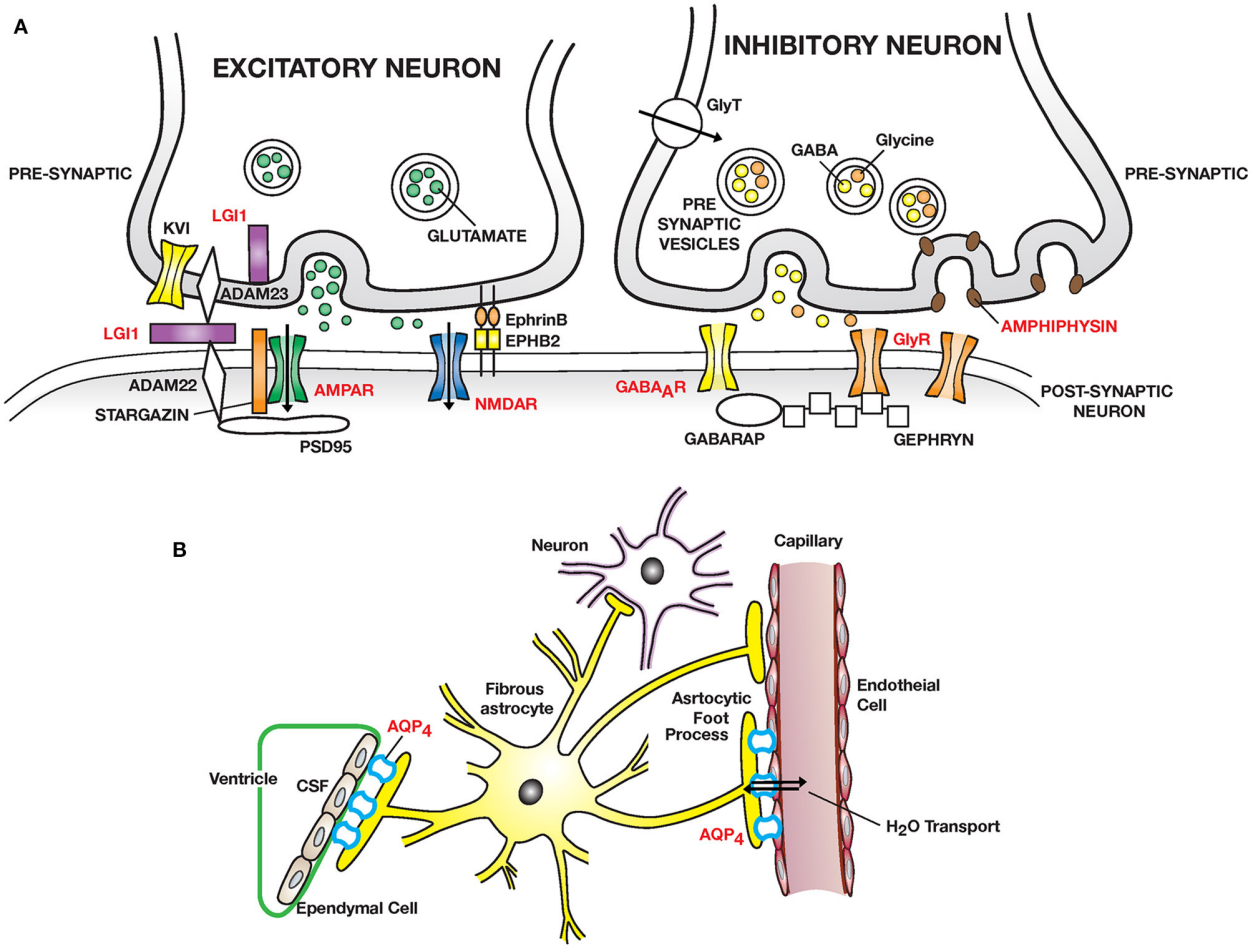
Autoimmmune diseases of the CNS: **(A)** Excitatory synapses: NMDAR encephalitis is caused by anti–NMDA receptor (NMDAR) antibodies binding and cross-linking GluN1 subunits, disrupting interaction of NMDAR with ephrin type B receptor 2 (EPHB2) causing internalization of NMDAR and impaired glutamergic transmission. LGI1 encephalitis is caused by auto-antibodies to leucine rich glioma inactivated protein 1 (LGI1), a neuronal glycoprotein secreted into the synapse. LGI1 interacts with presynaptic disintegrin and metalloproteinase domain-containing protein 2 (ADAM23) and postsynaptic ADAM22, modulates AMPA receptor trafficking and presynaptic Kv1, voltage gated potassium channel function. PSD95, post-synaptic density protein 95. Other receptors targeted: AMPAR; mGluR1/5. Inhibitory synapses: Autoantibodies to GABA type A receptor (GABA_A_R) on inhibitory synapses may lead to encephalitis with intractable seizures. Autoantibodies to the glycine receptor (GlyR) cause painful spasms; progressive encephalitis with rigidity and myoclonus (PERM). Stiff person syndrome can also be caused by auto-antibodies to amphiphysin, a presynaptic protein expressed in all synapses, important in clathrin mediated endocytosis leading to a decreased number of presynaptic vesicles filled with neurotransmitter available for exocytosis. Higher tonic activity may make inhibitory synapses (release of GABA and glycine) especially vulnerable. GABARAP, GABA associated receptor protein. GAD, decarboxylates glutamate to GABA (not shown). **(B)** Neuromyelitis optica (NMO) is caused by auto-antibodies to Aquaporin 4 (AQP4) water channel expressed widely in the body including on astrocytic foot processes abutting capillary endothelial cells and ependymal cells. Predominant pathology is in spinal cord, optic nerve and brain peri-ependymal regions.

**Figure 2:**
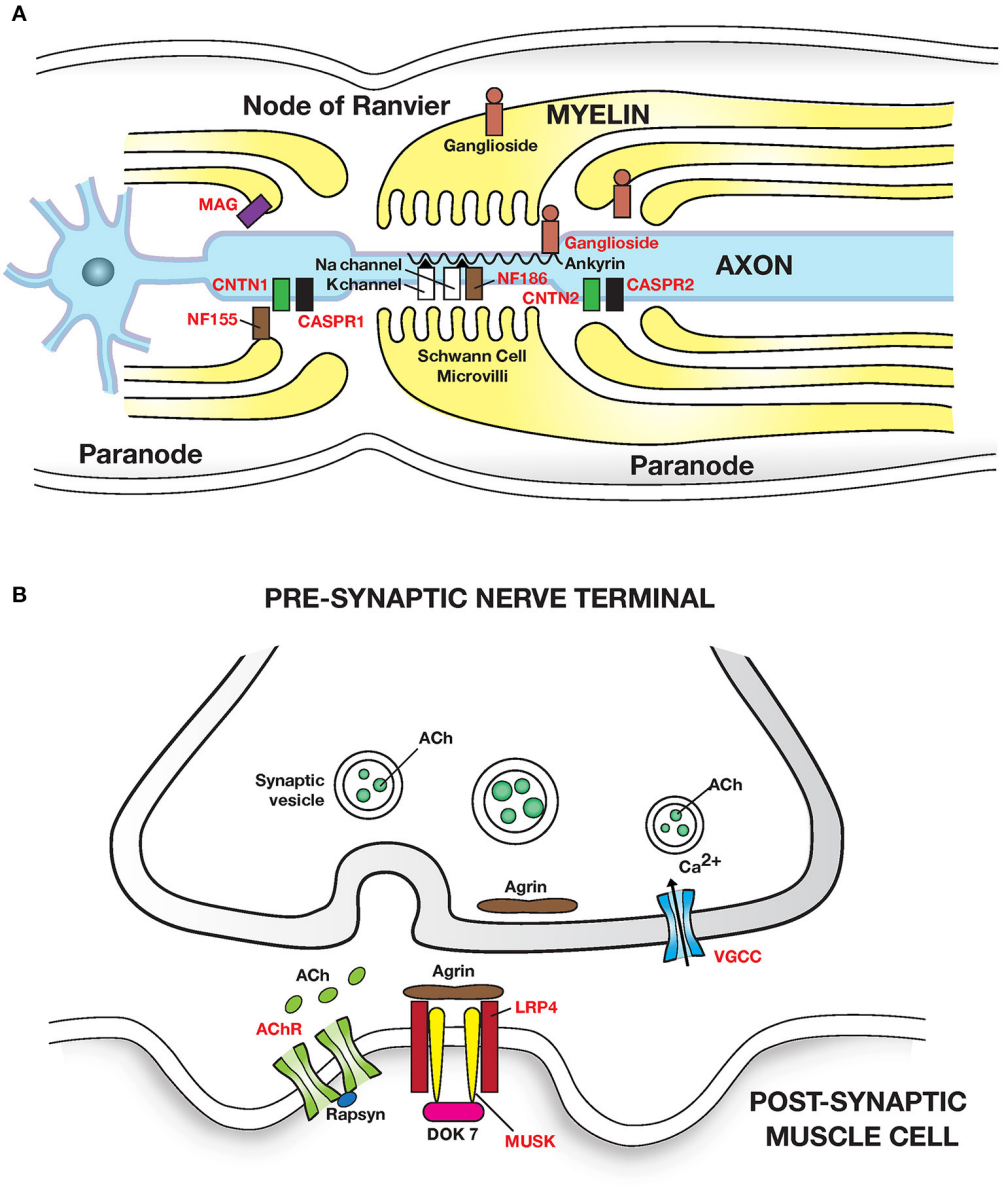
Autoimmune diseases of peripheral nerve and neuromuscular junction: **(A)** Peripheral nerve: Guillain Barre syndrome and its variants have been linked to autoantibodies to gangliosides (glycosphingolipids) which are abundant in peripheral nerves. Autoantibodies cause segmental demyelination and disruption of Na channel clustering. GM1 and GalNAc-GD1a ganglioside are most abundant in axolemma of motor nerves and nodes of Ranvier and are associated with motor phenotypes. GQ1b is enriched in oculomotor cranial nerves and is linked with Miller Fisher syndrome. Multifocal motor neuropathy (MMN) is linked to anti-GM1 ganglioside antibodies. MGUS neuropathy is linked to autoantibodies to myelin associated glycoprotein (MAG). Chronic inflammatory demyelinating polyneuropathy (CIDP) is linked to autoantibodies (in <10% patients) to the nodal and paranodal adhesion proteins neurofascin 186 and 155 (NF186, NF155); contactin 1 (CNTN1); contactin associated protein (CASPR1 and 2) which disrupt axon-Schwann cell contacts. Adhesion molecules are attached to the cytoskeleton by proteins such as Ankyrin/Spectrin. **(B)** Neuromuscular junction: Myasthenia gravis caused by autoantibodies to the (i) post synaptic Acetylcholine receptor (AChR); IgG1 and IgG3 autoantibodies activate the complement cascade leading to structural damage to postsynaptic membrane and loss of AChR. (ii) Muscle skeletal receptor tyrosine protein kinase (MUSK). MUSK autoantibodies (Fab- arm exchanged monovalent IgG4) block agrin (released from motor nerve terminals) induced MUSK—LRP4 (low density lipoprotein receptor related protein 4) interaction, and disrupt clustering of AChR. Dok7, Downstream of tyrosine kinase 7: cytoplasmic protein that activates MUSK in concert with agrin and LRP4. Lambert-Eaton myasthenic syndrome is caused by autoantibodies to presynaptic P/Q type voltage gated calcium channels (VGCC) disrupting Ca^2+^ influx; Ca^2+^ is essential for release of ACh from presynaptic vesicles. Antigenic targets of autoantibodies are shown in red text in all figures. Autoantibodies associated with inflammatory myopathies are shown in Table 3.

Important discoveries over the last two decades have identified many new autoimmune diseases of the nervous system and illuminated their pathogenesis. The clinical and pathophysiological features of CNS autoimmune diseases are outlined in Tables 1, 2 and Figure 1 and of the PNS in Table 3 and Figure 2 (44–50, 52, 62, 63, 67, 72).

**Table 2:** Classic and new CNS autoimmune diseases.

**Disease/target antigen**	**Auto-Ab pathogenic**	**Auto-Ab marker**	**Main pathological features**	**Main clinical features**
Multiple sclerosis/unknown	None Antibody to βsynuclein linked to gray matter changes	None	Complex autoimmune process, T and B cell involvement. Multifocal inflammation, prominent CD8^+^ cells, demyelination, oligodendrocyte loss, reactive gliosis, axonal degeneration	Optic neuritis, focal weakness, ataxia, myelopathy, opthalmoplegia, usually relapsing remitting
NMO spectrum disease/AQP4 water channel	AQP4 Mainly IgG1	Anti-MOG in some AQP4 negative	Necrosis, dystrophic astrocytes, granulocytic inflammatory infiltrate, loss of AQP4 reactivity, demyelination, perivascular IgG and complement deposits	Optic neuritis, transverse myelitis, area postrema (floor of fourth ventricle) involvement, intractable vomiting; secondary narcolepsy
ADEM/Unknown MOG in some	None	Anti-MOG Positive in ~40%	Widespread diffuse inflammation and demyelination, perivenular, mainly CD4^+^ T cells and granulocytic infiltration.	Usually children; acute encephalopathy, seizures, hemiparesis, ataxia, optic neuritis, myelitis Commonly follows 2–3 weeks after viral infection
MOG antibody-associated disease/MOG		Anti-MOG	Inflammatory demyelinating lesions of optic nerve, spinal cord, brain stem.	Optic neuritis: Bilateral, recurrent; optic disc edema common. Myelitis; ADEM; cranial nerve involvement
Narcolepsy/unknown	Unknown	None	Selective loss (>90%) of hypocretin producing neurons in hypothalamus. Evidence of genetic susceptibility (HLADQB1) and infectious trigger. Hypocretin-specific autoreactive CD4^+^ and CD8^+^ T cells detected in Narcolpesy patients blood and CSF. Anti-TRIB2 antibodies found in serum of 14 to 26% of narcolepsy patients (directed against protein produced in hypocretin neurons, Tribbles homolog2).	Excessive daytime sleepiness, cataplexy, hypnagogic hallucinations, sleep paralysis
Glial Fibrillar Acidic Protein (GFAP)		Anti_GFAP IgG	Meningo-encephalomyelitis May be associated with teratomas. Antibodies best detected in CSF.	Subacute to chronic meningitis—headache, neck stiffness, photophobia; Encephalitis: memory loss, tremor, ataxia; Myelitis: Usually mild—weakness, numbness, urinary retention; Bilateral optic disc edema Steroid responsive

*AQP4, Aquaporin-4 water channel; MOG, myelin oligodendrocyte glycoprotein; NMO, neuro-myelitis optica; ADEM, acute disseminated encephalomyelitis (62–68)*.

**Table 3:** Autoimmune diseases of the peripheral nervous system.

**Disease**	**Target antigen**	**Auto-antibody**	**Antibody frequency**	**Clinical features**	**Pathology**
**Autoimmune diseases of peripheral nerve**					
AIDP AMAN AMSAN Miller-Fisher	Unknown GM1, GD1a, GalNAcGD1aGM1, GD1a GQ1b, GT1a	None GM1, GD1a GalNAcGD1a GM1, GD1a GQ1b, GT1a	— 80% 90%	Acute ascending motor weakness, areflexia, facial weakness, autonomic changes, demyelinating neuropathy Predominantly axonal neuropathy Opthalmoplegia, ataxia, areflexia (With impaired consciousness and GQ1 antibodies suggests Bickerstaff brain stem encephalitis)	Inflammatory neuropathy with endoneurial/perivascular lymphocytic infiltrates; segmental demyelination in nerves, roots induced by complement fixing antibodies, macrophages Anti-ganglioside antibodies bind to nodes of Ranvier causing Na channel disruption
CIDP	Unknown in most patients NF155 NF140/186 CNTN1 Caspr1	Usually none NF155-IgG4 NF140/86 CNTN1-IgG4 Caspr1-IgG4	None in 80% 3–5% 1–2% 1–4% 1%	Chronic sensorimotor neuropathy AND: Tremor, sensory ataxia Aggressive course Sensory ataxia, glomerulonephritis Severe sensorimotor neuropathy	Chronic demyelination with predominantly macrophage infiltration of perineurium and endoneurium Antibodies target paranodal adhesion molecules and disrupt paranodal axon-Schwann cell contacts
Multifocal motor neuropathy (MMN)	GM1	Anti-GM1 antibodies	~50%	Slowly progressive, asymmetric, pure motor weakness, cramps, fasciculations, atrophy, areflexia, nerve conduction block common	Node of Ranvier or paranodal dysfunction. Possible immune mediated impairment of Na+/K+ ATPase pump function
MGUS neuropathy	MAG	Anti-MAG IgM	~50%	Sensory ataxia, distal weakness of foot and hand muscles	Demyelination of nerves with IgM and complement deposition
CANOMAD	GD3, GD1b, GT1b, GQ1b	IgM antibodies; Cold agglutinins	100% 50%	Sensory ataxic neuropathy, opthalmoplegia	Demyelination of sensory nerves, dorsal roots, minimal inflammation
Paraneoplastic polyneuropathy	HuD CasPR2 CRMP5 Amphiphysin	Anti-Hu, Anti-CasPR2 Anti-CRMP5 Anti-amphiphysin		Sensory neuronopathy, small fiber neuropathy, neuropathic pain, neuromyotonia	Usually associated with SCLC, NSCLC Thymoma ~20%
**Autoimmune diseases of the neuromuscular junction**					
Myasthenia gravis	α1subunit AChR MUSK LRP4	Anti-AChR IgG1, IgG3 Anti-MUSK IgG4 LRP4 IgG1,IgG2	~80%–Gen ~50%-Ocular 1–10% <5%	Proximal muscle weakness, fatiguability, ocular, bulbar, respiratory compromise More bulbar involvement Milder involvement	Loss of AChR, structural damage at post-synaptic membrane secondary to binding of AChR antibody, complement activation. Thymoma in 10–20%
Lambert-eaton myasthenic syndrome	α 1A subunit of pre-synaptic voltage gated Ca channels	Anti-VGCC Sox1	~60% paraneoplastic	Proximal muscle weakness, Autonomic: dry mouth, erectile dysfunction Decreased reflexes	Decreased Ca currents leads to decreased release of ACh from presynaptic vesicles. Usually SCLC
**Autoimmune diseases of skeletal muscle**					
Dermatomyositis	Mi2 NXP2 TIF-1 MDA5 SAE	Anti-Mi2 Anti-NXP2 Anti-TIF1 Anti-MDA5 Anti-SAE	Together found in 70% of patients	Mild muscle involvement with rash. Distal weakness, rash, calcinosis, dysphagia, increased cancer risk. Marked skin changes, mild or no myositis, strong cancer association. Severe skin rash, ulcers of palm, digits, minimal myositis, severe interstitial lung disease Mild/moderate myositis with skin rash	Perifascicular atrophy; inflammatory perivascular and perimysial infiltrate of B cells, macrophages, CD4+ T cells; MHC 1 upregulated on sarcolemma
Overlap myositis: anti-synthetase syndrome	Histidyl tRNA synthetase Threonyl tRNA synthetase Alanyl tRNA synthetase	Anti-Jo Anti-PL7 Anti-PL12		Mild/moderate myositis, interstitial lung disease, skin rash, Raynaud's phenomenon Similar to anti-Jo; more severe lung disease Mild myositis, severe lung disease	Perifascicular muscle necrotic fibers; Electron microscopy: Nuclear actin aggregation not seen in other inflammatory myopathies
Immune mediated necrotizing myopathy	SRP HMGCR	Anti-SRP Anti-HMGCR		Inflammatory myopathy, high muscle enzymes, dysphagia, lung involvement in 20% Severe myopathy with statin use	Muscle necrosis, regeneration, MHC1 upregulated

*AIDP, Acute inflammatory demyelinating polyneuropathy; AChR, acetylcholine receptor; AMAN, Acute motor axonal neuropathy; AMSAN, Acute motor and sensory axonal neuropathy; CANOMAD, chronic ataxic neuropathy with opthalmoplegia, monoclonal proteins, cold agglutinins and disialosyl antibodies; CIDP, Chronic inflammatory demyelinating polyneuropathy; CNTN1, contactin1; CASPR1, contactin associated protein 1; GM1, GQ1b, GD1a, GT1a etc., gangliosides, which are glycolipids located on axonal membranes or Schwann cells; HMGCR, 3hydroxy 3-methylglutaryl coenzyme A reductase; LRP4, lipoprotein receptor-related protein 4; MAG, myelin associated glycoprotein; MDA5, melanoma differentiation associated gene5; MHC, major histocompatibility complex; MMN, multifocal motor neuropathy; MGUS, monoclonal gammopathy of unknown significance; MUSK, muscle skeletal receptor tyrosine kinase; NF155/186, neurofascin splice variant 155/186; NXP2, nuclear matrix protein 2; TIF1: transcription intermediary factor1; SAE, small ubiquitin-like modifier activating enzyme; SRP, signal recognition particle (69–71)*.

### Nervous System Autoimmune Diseases With Intracellular or Neuronal Cell Surface Antigenic Targets

Autoimmune disorders of the nervous system may have: (1) Autoantibodies directed against intra-cellular neuronal antigens (classic paraneoplastic disorders) (47). The presence of these antibodies (such as anti-Hu, ant-Ri, and anti-Ma) is almost always associated with the presence of an underlying malignancy (Table 1). It is believed these disorders are caused by a misdirected immune response directed against tumor proteins (onconeural antigens) which are also expressed by neurons. But the auto-antibodies, which are aberrantly directed against intra-cellular neuronal antigens, have been shown in studies of live cultured neurons not to reach their intra-cellular targets and are not themselves pathogenic. They are useful, however, as bio-markers for these paraneoplastic disorders, which are caused mainly by T cell mediated pathology. This is supported by autopsy material which shows extensive infiltration of neurons by cytotoxic T cells, causing degeneration by perforin and granzyme B mediated mechanisms. Consequently patients with these paraneoplastic CNS disorders respond poorly to immunotherapeutic strategies involving removal of antibodies or antibody producing cells (Table 1); (2) Autoantibodies may be directed against neuronal cell surface and synaptic receptor proteins [(45), Table 1]. Cancer association in these disorders is less frequent (except in GABA_B_R and AMPAR encephalitis where cancer association is more common). Several recently described encephalitides (such as NMDAR, LGI1, and CASPR2 encephalitis) have been shown to be secondary to the pathogenic effect of autoantibodies binding to extracellular epitopes of neuronal cell surface proteins (Table 1). Imunotherapy to remove or neutralize the effect of these pathogenic autoantibodies is effective. Similarly, autoantibodies to cell surface proteins at the neuromuscular junction (acetylcholine receptors and voltage gated calcium channels) are pathogenic and result in myasthenia gravis or Eaton-Lambert syndrome. Immunotherapy has been shown to be effective in these disorders.

In addition to these two categories (intracellular or cell surface antigenic expression) a more complicated pattern of expression is illustrated by glutamic acid decarboxylase (GAD) an intracellular enzyme whose physiological function is the decarboxylation of glutamate to gamma-aminobutyric acid (GABA) the main inhibitory transmitter within neurons. GAD 65 is able to associate with the plasma membrane and surge to the extracellular space. Thus, epitopes of GAD65 and also amphiphysin can be exposed to antibodies during synaptic fusion and reuptake. High titers of anti-GAD65 have been linked to stiff person spectrum disorders, cerebellar ataxia and limbic encephalitis (73).

### Clinical Features Which Should Trigger Consideration of Autoimmune Diseases

The presentation of autoimmune encephalitis can be similar to encephalitis secondary to infections. Encephalitis is suggested by acute or subacute symptoms of decreased or altered level of consciousness, lethargy, short term memory loss, personality changes such as apathy, irritability, agitation and commonly, seizures.

The following features (developing over days to weeks not years), usually in patients with suspected encephalitis, should trigger consideration of autoimmune causes:

• After excluding infectious causes, acute or sub-acute development of cognitive decline, behavioral changes and seizures (typically intractable and resistant to anti-epileptic medications) should prompt consideration of autoimmune encephalitis. Seizures occur most commonly in autoimmune encephalitis with NMDAR, LGI1, and GABA_B_R antibodies. When this clinical presentation is associated with bilateral medial temporal lobe abnormal signal on MRI FLAIR images, limbic encephalitis should be considered. Although limbic encephalitis can also be caused by CNS infections such as HSV or HHV6 encephalitis, when autoimmune in etiology the usual antibodies associated with limbic encephalitis are anti-Hu, Ma2, LGI1, GABA_B_, or AMPA receptor.
• Faciobrachial dystonic seizures characterized by brief (few seconds), lateralized (unilateral or bilaterally asynchronous) tonic contractions mainly affecting the upper limb and face often associated with hand dystonia. Episodes can recur numerous times a day and are considered pathognomic of anti-leucine-rich glioma inactivated 1 (LGI1) encephalitis (74).
• Prominent, recent, psychiatric manifestations including agitation, aggression and violence, visual or auditory hallucinations, delusions, disorganized or bizarre behavior, paranoia, disinhibition, apathy, mutism and catatonia; intolerance to neuroleptics may cause hyperthermia, rigidity, rhabdomyolysis or coma. Such features have most typically been described with NMDAR encephalitis (in up to 95% of patients) (75, 76).
• Cerebellar ataxia (pan cerebellar involvement) presenting subacutely (<3 months) as an isolated symptom or associated with limbic or brain stem encephalitis, myelitis or neuropathy. It is associated with multiple antibodies, such as anti-Hu (ANNA-1), mGluR1, CASPR2, GAD65, Ri (ANNA-2), Yo (PCA1), PCA2, CV2/CRMP5, or Sox1. Opsoclonus-myoclonus in an adult in isolation or together with cerebellar ataxia may be associated with anti-Ri antibodies. Decreased level of consciousness, bilateral external opthalmoplegia and ataxia developing over <4 weeks suggests Bickerstaff brainstem encephalitis and is associated with antibodies to anti-GQ1b antibodies.
• Sleep disorders such as abnormal sleep architecture with undifferentiated NREM sleep, REM sleep behavior disorder (RBD), obstructive sleep apnea and stridor, and agrypnia excitata (insomnia, motor, and autonomic hyperactivation). Various features of sleep disorders and RBD have been described in patients with antibodies to IgLON5, CASPR2, Ma2, and LGI1 (77). NMOSD with hypothalamic involvement can present with secondary narcolepsy.
▪ Movement disorders such as chorea, dyskinesias, dystonias, or myoclonus (associated with multiple antibodies including anti- NMDAR, CASPR2, LGI1,Ri, Hu, or CV2/CRMP5); and rarely parkinsonism (associated with anti-Ri, NMDAR, LGI1, and D2R) (78).
• Peripheral nerve hyperexcitability (neuromyotonia) presenting as spontaneous motor activity with cramps, myokimia or fasciculations which may be associated with encephalopathy (associated with anti-CASPR2 and LGI1 antibodies).
▪ Stiff person spectrum disorders presenting as fluctuating muscle spasms and rigidity and acquired hyperekplexia (startle response) is associated primarily with antibodies that target proteins expressed by inhibitory synapses such as glutamic acid decarboxylase 65 (GAD65), α1 subunit of the glycine receptor (GlyR) or amphiphysin (79–81).
• Autonomic disturbances such as hyperthermia, fluctuations of blood pressure, tachycardia, less commonly bradycardia requiring a temporary pacemaker, excessive salivation, hyperhidrosis, chronic gastro-intestinal pseudo-obstruction and central hypoventilation. These have typically been described in patients with anti- NMDAR and anti-Hu encephalitis.
• Sensory neuronopathies are a rare group of disorders secondary to degeneration of dorsal root ganglion presenting with asymmetric, multifocal, non-length dependent sensory loss, neuropathic pain and subacute sensory ataxia secondary to proprioceptive loss. This syndrome, in isolation or often together with encephalomyelitis, has been linked to anti-Hu, CV2/CRMP5 and amphiphysin antibodies in cancer patients.
• Multifocal encephalitic presentation (in children and young adults) characterized by multiple, large white matter and deep gray matter demyelinating lesions on brain MRI scans is suggestive of acute disseminated encephalomyelitis (ADEM). MOG antibodies may be present in serum in up to 50 per cent cases.

Unilateral or bilateral optic neuritis presenting as sudden visual loss may be associated with antibodies to AQP4 (NMO spectrum disease) or MOG (MOG antibody- associated disease) or multiple sclerosis (usually unilateral) (82) (Table 2). Myelitis presenting as rapid onset of paraparesis or quadriparesis may be associated with AQP4 antibodies (NMO spectrum disease); MOG antibodies (MOG antibody- associated disease) (83, 84); MS or ADEM.

### Tests Done for Initial Evaluation (85, 86)

(1) CSF analysis in autoimmune encephalitis may be normal or non-specific with mild lymphocytic pleocytosis and elevation of CSF protein; (2) MRI brain studies in autoimmune encephalomyelitis may be normal or may show scattered white matter lesions on T2 weighted FLAIR images. Autoimmune limbic encephalitis is characterized by abnormal signal in bilateral medial temporal lobes; in NMO there may be swollen, enhancing optic nerves, longitudinally extensive (> 3 vertebral segments) spinal cord lesions with increased T2 signal with enhancement, and peri-aqueductal and dorsal medulla/pons lesions; (3) EEG findings are usually non-specific with focal or diffuse slowing with or without epileptogenic activity. In NMDAR encephalitis an unique EEG pattern of 20–30 Hz beta activity riding on rhythmic 1–3 Hz delta waves, called extreme delta brush has been described. In primary psychiatric or functional disorders MRI Brain, EEG and CSF analysis will be normal providing an useful initial assessment for the presence or absence of structural neurologic disease; (4) CT Chest, abdomen and pelvis with and without contrast should be done for tumor screening; consider pelvic/vaginal ultrasound for ovarian tumors and FDG-PET/CT scan for whole body tumor detection when clinical suspicion is high and other tests are non-revelatory; (5) PNS disorders are typically assessed by nerve conduction, repetitive stimulation and electromyographic (EMG) studies and creatine kinase (CK) estimation. Nerve or muscle biopsies may sometimes be useful.

### Autoantibody Testing

Both serum and CSF should be tested for IgG autoantibodies. CSF should also be tested as CSF neuronal antibodies are more disease specific than serum antibodies and antibodies are only present in CSF in some cases. For example, in NMDAR encephalitis, no serum anti-NMDAR antibodies are found in ~15% of patients. It should be noted that conversely, in ~10% of patients of NMOSD and LGI1 encephalitis, antigen-specific autoantibodies can only be detected in serum, not in CSF (27). Auto-antibodies in the classic paraneoplastic disorders (e.g., anti-Hu, anti-Ri) are more specific or only detected in serum. Similarly only a minority of ant-MOG-seropositive patients have detectable antibodies in CSF (87).

Antibody screening methodology is usually indirect immunofluorescence or immunohistochemistry or radioimmunoassay but definitive confirmation may require a cell based assay or Western blot assay. For classic paraneoplastic disorders the first screen is by Western blot and cell based assays are often used from the outset if the clinical suspicion is high for autoimmune encephalitis. Most commercial laboratories test for panels of autoantibodies as similar presentations may underlie the effect of different auto-antibodies (Table 1). When a characteristic clinical presentation such as faciobrachial dystonic seizures are present a single antibody, LGI1 can be tested at the outset. Determination of immunoglobulin class is important as IgM or IgA antibody positivity may be seen in healthy controls and caution should be exercised before considering them clinically significant. Antibody positivity should be considered in the appropriate clinical context as low titers may be misleading. In NMDAR encephalitis high antibody titers correlate with a poor outcome (88), but this correlation is imperfect and antibody positivity may persist after recovery. Clinical assessment should therefore be used to guide treatment (89). Tests for CSF oligoclonal bands and CSF IgG index should be done as an indication of intra-thecal synthesis of antibodies.

### Important Role of B Cells in Autoimmune Diseases of the Nervous System

Both T and B cells play important roles in the pathogenesis of various autoimmune inflammatory nervous system disorders (90). The predominant role of B cells in some of these disorders has recently been emphasized (90, 91). B cells and their secreted antibodies recognize distinct antigens through a rearranged B cell receptor (BCR). Naïve B cells arise from hematopoietic stem cells in the bone marrow and circulate in peripheral blood and lymphoid organs. Following recognition of their cognate antigen in germinal centers a subset may eventually become CD27^+^ memory B cells, which can further differentiate into CD38^+^ antibody secreting plasmablasts and long lived CD138^+^ plasma cells. In addition to antibody production, B cells can internalize proteins and present peptide fragments to-antigen-specific CD8^+^ and CD4^+^ T cells in association with MHC Class I and Class II molecules, thus modulating T cell function. Current evidence suggests that B cell antigen presentation plays an important role in T cell activation in MS (91). B cells also produce a number of pro-inflammatory cytokines lymphotoxin-α, interleukin 6 and tumor necrosis factor or anti-inflammatory cytokines such as IL-10 and IL-35, thus having the ability to either promote or suppress CNS inflammation.

Recent work has suggested that changes in B cell development in the bone marrow, secondary to altered B cell intrinsic signals can lead to an altered naïve B cell repertoire and the generation of autoantibody producing B cells (92). These signals consist of a complex interplay of BCR signaling and co-receptor signaling, mediated by B cell activating factor (BAFFR), CD40 and Toll-like receptors (TLRs). Such altered signaling programs (secondary to genetic variants associated with autoimmunity) could result in the production of a higher proportion of mature B cells which exhibit autoreactivity (92).

## Illustrative CNS Autoimmune Disorders

### Multiple Sclerosis

Although in MS the autoimmune response to unknown target antigens has traditionally been considered to be driven by T cells, the importance of B cells in pathophysiology has been highlighted by, (1) the detection of CSF oligoclonal bands, unique IgG fractions produced by an inthrathecal clonal B cell population which target ubiquitous self-antigens; (2) Detection of B cells in CNS lesions in MS, most abundantly in active lesions; (3) ectopic lymphoid follicle like structures containing CD20^+^ B cells, CD138^+^ plasma cells and follicular dendritic cells, which have been identified in the leptomeninges of patients with MS; (4) expanded populations of plasmablasts and plasma cells obtained from patients with MS, which target neurons, astrocytes and oligodendrocytes, although the target antigen is unknown; (5) the detection of CSF antibody reactivity against measles, rubella and varicella-zoster viral antigens (93). Collectively, it has been proposed that this data suggests that the humoral B cell response in MS may not be directed against a single antigen but may be directed against a wide array of self and non-self antigens which could differ from individual to individual (91). The broad range of targets may reflect epitope spreading and secondary immune reactions to CNS damage.

The remarkable efficacy of therapies which target CD20 in MS patients has confirmed the critical role of B cells in MS pathogenesis. Interestingly the clinical benefit of anti-CD20 B cell therapy in MS precedes reduction in total IgM and IgG levels, demonstrating that the effect is not due to a reduction in humoral immunity but may be secondary to impaired antigen presentation to T cells, or decrease in inflammatory cytokines such as IL-6 or TNF. Furthermore, the most effective therapies for MS involve the ablation or blocking the trafficking of B and T cells in the periphery, which results in a substantial reduction in new CNS lesions and relapses. This suggests that MS relapses are mediated by episodes of BBB breakdown with recruitment of peripheral lymphocytes and macrophages into the CNS (91). Thus, the lack of efficacy in some types of MS, such as in progressive MS, may be secondary to sequestration of the abnormal immune response behind an intact blood brain barrier.

### NMO Spectrum Disease

NMOSD is an inflammatory demyelinating lesion primarily involving the optic nerves, spinal cord and brainstem. Anti–Aquaporin 4 (a membrane bound water channel expressed abundantly on astrocytic foot processes)-IgG is present in 70–90% of NMO patients. The pathogenicity of anti-AQP4 antibodies has been demonstrated both *in vitro* and *in vivo* and intracerebral injection of AQP4 IgG with complement results in demyelination resembling NMOSD. Serum titers of anti-AQP4 antibodies are 1,000-fold higher than in CSF and CSF oligoclonal bands, which are seen in only 15–30% of NMO patients usually disappear with disease progression, implying that B cell activation and the origin of the humoral immune response is outside the CNS. Activated B cells may then enter the CNS after BBB breakdown and cause pathology, as discussed earlier.

During normal early B cell development, tolerance is maintained by the removal of most self-reactive B cells. Anti-AQP4 IgG producing B cells seem to arise from defects in early B cell tolerance checkpoints, both centrally in the bone marrow and peripherally, resulting in an increased number of autoreactive B cells in the mature naiive B cell population (94). This reservoir of auto-reactive B cells can supply B cell clones which produce pathogenic anti-AQP4 antibodies after somatic hypermutations. This conclusion has been based on the observation that the unmutated precursors of B cells secreting anti-AQP4 antibodies do not bind to autoantigen, showing that anti-AQP4 specificity and the generation of pathogenic autoantibodies require affinity maturation and the acquisition of somatic hypermutations, a process regulated by T cells (94). Additionally, the activation of B cell clones may initially be triggered by an antigen other than AQP4, such as to a peptide from Clostridium perfringens, an indigenous intestinal bacterium (which has homology to an immunodominant epitope of AQP4).

### Myelin Oligodendrocyte Glycoprotein—Antibody Associated Disease

MOG is produced by oligodendrocytes, the myelin forming cells of the CNS. It is expressed on the surface of oligodendrocytes and the external lamella of myelin sheaths. It has been of great interest historically in the study of MS as it had been shown to be the target antigen in an animal model of MS, experimental allergic encephalomyelitis (95). However, studies in MS showed that MOG antibodies were non-specific (96). It has only been recently with better antibody detection methods using cell based assays, which detect MOG in its native conformational state, that MOG-antibody has emerged as a biomarker for CNS inflammatory demyelinating disorders distinct from MS and NMOSD. A recent study has confirmed that the most reliable assays to detect MOG antibody are those using live cells expressing native full length human MOG (as some conformational epitopes are lost on fixation of MOG expressing cells) (64). Secondly, low positive samples are detected widely, including in healthy individuals, therefore an appropriate cutoff value to identify the clinically meaningful results is an area requiring further studies. Serum testing is recommended for detecting MOG-IgG as the reliability of CSF MOG-IgG is uncertain. Also oligoclonal bands are infrequently detected in CSF, unlike MS, implying the peripheral initiation of the autoimmune process.

In MOG-antibody associated disease preceding prodromal symptoms are commonly encountered and can include fever, malaise, cough and rhinorrhea (65). The majority of patients present with optic neuritis, transverse myelitis or acute disseminated encephalomyelitis (ADEM, which is most prevalent below the age of 20 years) (66). Brain stem or cerebellar involvement presenting with ataxia, facial palsy, diplopia or vertigo may be seen (97). A recent study has also reported the occurrence of overlapping central and peripheral nervous system involvement (acute inflammatory demyelinating neuropathy, myeloradiculitis, multifocal motor neuropathy or brachial neuritis) (98). Clinical course may be either monophasic or relapsing. In ADEM, high titers and persistent MOG-IgG positivity predict a higher risk of relapse (83). Optic neuritis can be unilateral or bilateral and is often associated with optic disc edema. MRI may show enhancement along the length of the optic nerve including the optic sheath. Bilateral anterior optic nerve enhancement without extension to the optic chiasm is typical (82). In patients presenting with myelitis on MRI, longitudinally extensive lesions occur in the majority, with involvement of the conus seen more commonly than in NMOSD. Spinal cord lesions on MRI typically involve the gray matter, distinguishing them from lesions in MS and NMOSD (84).

### NMDAR Encephalitis

NMDAR encephalitis is the commonest autoimmune encephalitis, characterized by IgG auto-antibodies directed against the GluN1 subunit of the NMDAR (99). The disease shows a female predominance (female to male ratio of 8:2) with a median age at presentation of 21 years (range 1–85 years) and is commonly associated with ovarian teratoma. The most common presentation is abnormal behavior (visual or auditory hallucinations, acute schizoaffective disorder, depression, mania), cognitive dysfunction, seizures, movement disorders (oral, facial, lingual dyskinesias, chorea, athetosis, and dystonia), autonomic dysfunction or central hypoventilation. Most (80%) patients recover after immunotherapy and ovarian teratoma removal (if present). Diagnosis is confirmed by the presence of NMDAR IgG antibodies in cerebrospinal fluid. Brain biopsy or autopsy shows infiltrates of B cells, CD4+ T cells and deposits of IgG but little or no neuronal loss, providing an explanation for the significant recovery that can be achieved after early immunotherapy.

Multiple lines of evidence show that NMDAR antibodies in patients serum are pathogenic (100). In experiments involving cultured neurons, antibodies crosslink NMDARs, cause their internalization and impairment of synaptic plasticity (100). In animal experiments, infusion of patient CSF or IgG purified from patient CSF into the ventricular space of mice via intraventricular catheters leads to memory deficits, anhedonia and seizures (101–103). Furthermore, active immunization of mice with GluN1/GluN2B heteromers results in a fulminant encephalitis (104). The NMDAR antibodies produced cause a reduction of NMDAR and NMDAR currents in cultured neurons. Also transplacental transfer of NMDAR antibodies from pregnant patients to embryos results in neurological deficits in neonates (105, 106). Overall these findings support the role of NMDAR autoantibodies in disease causation as required by Witbesky's postulates.

Unlike NMOSD (where antibodies are much more readily detected in serum than in CSF), anti-NMDAR antibodies are more reliably detected in the CSF than in serum (both should be tested). Also, clonally expanded NMDAR-specific plasma cells have been detected in the CSF of patients with NMDAR encephalitis and B cells in the CSF of these patients can produce anti-NR1 antibodies. Taken together, in contrast to myasthenia gravis and NMOSD, in which antibody production occurs in the periphery, this data suggests that the NMDAR specific B cell response in anti-NMDAR encephalitis is compartmentalized in the CNS.

### Checkpoint Inhibitor Therapy Induced Nervous System Autoimmune Disease

An uncommon but intriguing autoimmune disorder has recently been described (107). Inhibitory molecules (such as CTLA-4, PD-1, LAG-3, and TIM3) which are expressed on the surface of T and B lymphocytes inhibit and keep a check on excessive immune responses both normal and anti-self. Immune checkpoint inhibitors are a new treatment for cancer which is being used more commonly in recent years. They usually target programmed death 1 (PD-1) or cytotoxic T-lymphocyte antigen-4 (CTLA-4) expressed on T cells including T_reg_ cells. Recently 4 patients were described who developed transverse myelitis, Guillain Barre syndrome or myasthenia gravis 4 weeks after medication (Pembrolizumab or Nivolumab and Iplimumab) administration. Conventional auto-antibodies associated with these conditions were absent but serum immunoglobulins from the myelitis and Guillain Barre syndrome patient showed a novel pattern of tissue reactivity, binding to rodent brain tissue or myelinating stem cell derived sensory neurons or rat primary Schwann cells. It was suggested that the short time between drug administration and disease onset implies that T and B cells which were circulating in a quiescent state, were disinhibited leading to the activation of pre-existing B cell reactivity to neural and neuromuscular proteins (107).

## Illustrative PNS Autoimmune Disorders

Autoimmune disorders of the neuromuscular junction include myasthenia gravis (with auto-antibodies directed against the post-synaptic Acetylcholine receptor or muscle specific tyrosine kinase) or Lambert-Eaton syndrome with autoantibodies directed against pre-synaptic voltage gated calcium channels. The definitive demonstration of the autoimmune basis of myasthenia gravis is one of the classic examples of an autoimmune disease (108, 109).

Although not as definitive, considerable indirect evidence is supportive of an autoimmune basis for some disorders of the skeletal muscle and peripheral nerve.

### Immune-Mediated Myopathies

Immune-mediate myopathies are a group of acquired muscle disorders which include dermatomyositis, necrotizing autoimmune myopathy, overlap myositis, and inclusion body myositis (Table 3) (69, 70). A good response to immunosuppressive therapy is seen in these disorders except in inclusion body myositis (see below). *Dermatomyositis* typically presents with proximal muscle weakness and a skin rash. Muscle biopsies show perifascicular atrophy with perimysial and perivascular infiltrates of CD4+ T cells, B cells and plasmacytoid dendritic cells with sarcolemmal upregulation of MHC1. The deposition of membrane attack complex on intramuscular capillaries is an early manifestation of dermatomysositis, but the primary cause of the microangiopathy remains unclear. About 70% of dermatomyositis patients have a specific autoantibody which are associated with unique clinical phenotypes (Table 3). For example the presence of anti-transcriptional factor 1α (TIF-1) or anti-nuclear matrix protein NXP2 antibodies are associated with a high risk of cancer. Patients with antibodies to melanoma differentiation associated gene 5 (MDA5) have severe cutaneous changes with skin ulcers and interstitial lung disease. *Overlap myositis*, typically represented by anti-synthetase syndrome, is a type of autoimmune myopathy associated with other connective tissue diseases. These patients have auto-antibodies to various aminoacyl tRNA synthetases (Table 3). Patients with anti-Jo antibodies have myopathy associated with interstitial lung disease, radial finger skin lesions called mechanic's hands and Raynaud's phenomenon. *Immune mediated necrotizing myopathy* is characterized by proximal muscle weakness with very creatine kinase levels and muscle biopsies showing necrosis and regeneration with minimal or no lymphocytic infiltration, upregulation of MHC 1 and membrane-attack complex deposition on non-necrotic fibers. About two-thirds of patients have autoantibodies to either signal recognition particle (anti-SRP) or 3-hydroxyl 3-methylglutaryl coenzyme-A reductase (HMGCR), an enzyme that catalyzes the rate limiting step in cholesterol synthesis. Antibodies to HMGCR are usually seen in patients who have been exposed to statins. *Inclusion body myositis* patients have evidence of CD8+ inflammatory infiltrates but also rimmed vacuoles, ragged red fibers indicating mitochondrial damage and abnormal protein aggregates on muscle biopsies suggesting both an inflammatory and a degenerative process. Auto-antibodies recognizing cytosolic 5'-nucleotidase 1 A are present in 30–60% of patients with sporadic inclusion body myositis. However, these are also found in 10–20% of patients with dermatomyositis, SLE and Sjogren's syndrome. The role of these autoantibodies in pathogenesis and in diagnosis requires further investigation.

### Immune-Mediated Neuropathies

Immune mediated neuropathies typically include acute inflammatory demyelinating polyradiculoneuropathy (AIDP; Guillain Barre syndrome), chronic inflammatory demyelinating polyneuropathy (CIDP), multifocal motor neuropathy (MMN) and neuropathies associated with IgM monoclonal gammopathy of undetermined significance (MGUS) (Table 3) (71). It is believed that autoimmune peripheral nerve damage is mediated both by antibodies directed against myelin antigens and autoreactive T cells and macrophages that invade myelin sheath or axonal membranes. Autopsies from Guillain Barre syndrome patients have shown perivascular and endoneurial inflammatory infiltrates of T cells and macrophages throughout the nerves, roots or plexuses along with segmental demyelination mediated by macrophages (110). Autoantibodies to various gangliosides which are abundant in axons are found commonly in patients (in 80–90%) with the Miller-Fisher subtype (GQ1b, GT1a) or the axonal variant (GM1, GD1a) of Guillain-Barre syndrome but very infrequently in the most common demyelinating subtype. In 15–20% of CIDP patients auto-antibodies have been detected in serum to several proteins at the nodal or paranodal regions of peripheral nerves (such as CASPR1, contactin1, and NF155) which maintain axon-Schwann cell or axon-myelin binding. These autoantibodies could activate complement and macrophage mediated axonal degeneration, demyelination and disruption of nodal architecture.

Paraneoplastic neuropathies are commonly sensory neuronopathies secondary to dorsal root ganglion pathology associated with anti-Hu, CRMP5 or amphiphysin antibodies. Peripheral nerve hyperexcitability and neuropathic pain may be seen with CASPR2 antibodies. Enteric autonomic involvement with severe disturbances of gastrointestinal mobility has been described with anti-Hu antibodies. Treatment of these disorders usually involves plasmapheresis, IVIG, and/or immunosuppression.

## Therapy

Current therapies for CNS and PNS autoimmune diseases and mechanism of action of various treatment modalities are detailed in Table 4.

**Table 4:** Therapy of autoimmune diseases of the nervous system.

**Main therapy**	**Mechanism of Action**
Multiple sclerosis**:** **Acute attack:** High dose (1 gram) Intravenous Methylprednisolone for 3–5 days. **Disease modifying agents:** Interferon-1b; Glatiramer acetate; Fingolimod; Teriflunomide; Dimethyl fumarate **High efficacy treatment:** Rituximab Ocrelizumab Alemtuzumab Natalizumab Mitoxantrone **Immune reconstitution:** Autologous hematopoietic stem cell transplantation (AHSCT)	**Fingolimod:** Binds to Sphingosine-1-phosphate receptors (to four of five receptors, SIPR1-5) and prevents egress of lymphocytes from lymph nodes into the circulation. **Siponimod:** binds selectively to SIPR1 and SIPR5. **Dimethyl fumarate:** Uncertain. Inhibits T cell proliferation; induces oxidative stress in T cells; inhibits antigen presenting capacity *in vivo*. **Terifluonamide:** selectively inhibits dihydro-orotate dehydrogenase, a key mitochondrial enzyme in the *de novo* pyrimidine synthesis pathway, leading to a reduction in proliferation of activated T and B lymphocytes. **Rituximab:** Chimeric mAb depletes naïve and memory B cells by targeting CD20 expressed on pre-B and B cells but not plasmablasts and differentiated plasma cells. **Ocrelizumab:** Humanized mAb depletes B cells by targeting CD20 (different but overlapping epitope compared to Rituximab). **Ofatumumab:** Human mAb targeting CD20, depletes B cells. **Alemtuzumab:** Humanized mAb targets CD52 expressed on all T, B lymphocytes, monocytes but not hematopoietic stem cells. Causes profound lymphopenia which recovers over 1–3 years (B cells before T cells). **Natalizumab:** Binds α4β1 integrin on leucocyte surface blocking interaction with VCAM-1; prevents transmigration of leucocytes across endothelial surface into CNS
Autoimmune encephalitis Treatment of cancer if present **First line:** High dose gluco-corticoids, plasmapheresis, IVIG **Second line:** Rituximab Tocilizumab Bortezomib Consider cyclophosphamide	**Plasmapheresis** (5–7 therapeutic plasma exchanges) is based on the separation of blood's cellular elements from plasma usually with a permeable filter 0.2 μm in diameter allowing removal of substances such as immunoglobulins (including autoantibodies), complement, immune complexes and endotoxins. Discarded plasma is replaced with fresh frozen plasma or albumin and together with blood cellular elements reinfused into patient. Reduction of autoantibodies has been noted in both serum and CSF, possibly through an inflamed blood-brain barrier with abnormally increased permeability (as antibodies cannot cross the normal blood-brain barrier). **Bortezomib:** Proteasome inhibitor causes apoptosis and depletes short lived and terminally differentiated long lived plasma cells (which are resistant to immunosuppression).
Neuromyelitis optica **Acute attack:** High dose Intra-venous methylprednisolone for 5 days. Plasmapheresis: 5–7 sessions. **Maintenance therapy:** Rituximab, mycophhenolate mofetil, azathiorine **Newer agents:** Eculuzimab, inebiluzumab, satralizumab, tocilizumab **Refractory patients:** AHSCT	**Eculizumab:** Humanized mAb that inhibits the terminal complement protein C5 and prevents cleavage into pro-inflammatory C5a and C5b. **Satralizumab, tocilizumab:** Monoclonal antibodies which binds the IL6 receptor and blocks the inflammatory cascade including decreasing differentiation of B cells into anti-AQP4 producing plasmablasts. **Inebiluzumab:** Monoclonal antibody that binds to the B cell surface antigen CD19, leading to cytolysis of plasmablasts and plasma cells and decreased auto-antibody production.
Guillain-Barre (AIDP)**:** IVIG; Plasmapheresis *Corticosteroids ineffective* CIDP**:** Glucocorticoids; IVIG; Plasmapheresis Refractory: rituximab Multifocal motor neuropathy**:** IVIG *Plasmapheresis and corticosteroids ineffective* Anti-MAG neuropathy**:** Usually refractory. Rituximab may be of some benefit	**IVIG:** (i) contains anti-idiotypic antibodies (antibodies directed against neutralizing Fab2 region of autoantibodies); (ii) Fab2 region of IgG in IVIG binds and inactivates pro-inflammatory C3b, C4b, C3a, C5a; (iii) IVIG saturates FcRn, a transport receptor for IgG on vascular endothelial cells that binds and protects pinocytosed IgG in endosomes. This normal protective effect of the FcRn receptor on IgG catabolism is lost, leading to increased IgG degradation and decreased auto-antibody levels; (iv) IVIG upregulates inhibitory FcλRIIb receptors on monocytes, macrophages and B cells, heightening their activation threshold and leading to decreased inflammation. *Patients with atypical CIDP phenotypes with IgG4 antibodies are resistant to conventional immunotherapy (IVIG and plasmapheresis)*.
Inflammatory myopathies**:** Glucocorticoids Methotrexate, azathioprine; mycophenolate mofetil cyclophosphamide; cyclosporine; tacrolimus; IVIG Refractory with severe lung disease: rituximab	**Corticosteroids:** Inhibits B cell proliferation and B cell receptor triggered signaling, plasma cell differentiation and immunoglobulin gene expression. Inhibits genes such as IL2 and the transcription factor GATA 3, leading to reduced differentiation of naïve T cells into T helper cells and reduces T cell activation of auto-reactive B cells.
Myasthenia gravis**:** **Symptomatic:** Pyridostigmine; **Acute attack:** IVIG; Plasmapheresis **Maintenance:** Thymectomy; glucocorticoids; **Steroid sparing:** Azathioprine; cyclosporine, methotrexate, mycophenolate mofetil **Refractory:** Rituximab Eculizumab; Cyclophosphamide	**Pyridostigmine:** Blocks Acetylcholinesterase action in synapse, decreasing metabolism of Ach leading to increased ACh levels. **Eculizumab:** Humanized mAB that inhibits the terminal complement protein C5 and prevents cleavage into pro-inflammatory C5a and C5b.
Eaton-lambert syndrome**:** **Symptomatic:** Amifampridine phosphate **Acute attack:** IVIG; Plasmapheresis **Maintenance:** Glucocorticoids, azathioprine. Treatment of associated cancer if present	**Amifampridine:** Blocks presynaptic K channels, prolongs action potential leading to longer duration of Ca channel opening and higher Ca++ levels resulting in increased release of ACh from pre-synaptic vesicles into synaptic cleft.

*ACh, acetylcholine; IVIG, intravenous immunoglobulin; AHSCT, Autologous haemopoietic stem cell transplantation; mAb, monoclonal antibody; IgG, Immunoglobulin G; IL6R, Interleukin 6 receptor; VCAM1, Vascular cell adhesion molecule 1*.

### Management of Autoimmune Encephalitis

Best practice recommendations for the management of autoimmune encephalitis have recently been made (111). If cancer is present it should be treated promptly as early treatment is associated with better prognosis. For example ovarian teratoma removal in NMDAR encephalitis is associated with expedited recovery (112). Typically patients are given immunosuppressive therapy which should also be started as early as possible as this is associated with a better prognosis (112). First line immunotherapy includes high dose intravenous methylprednisolone for 5 days. This is usually followed by plasmapheresis (5–7 exchanges) and/or intravenous immunoglobulins (IVIG). Second line therapy for non-responders may include IV Rituximab and Cyclophosphamide. The IL6 blocker tocilizumab may be beneficial in special circumstances such as refractory status epilepticus in these patients. In more severe cases 3–6 months of Cyclophosphamide may be added. Cyclophosphamide is often considered in the classic paraneoplastic disorders given its effectiveness in depleting T cells. Bortezomib, a proteasome inhibitor which targets long lived auto-reactive plasma cells can be a useful adjunct to standard immunotherapy in treatment-refractory patients (113).

The extent of recovery varies according to the type of encephalitis. In a large study of 577 patients with NMDAR encephalitis, after immunotherapy, 53% improved within 4 weeks and 81% improved by 2 years (112). Although 47% failed first line immunotherapy, second line immunotherapy was still found to be effective. Outcomes were found to continue to improve for up to 18 months after symptom onset (112). In LGI1 encephalitis, a study of 103 patients showed a striking time-sensitive response to early immunotherapy, with 51% showing cessation of faciobrachial dystonic seizures within 4 weeks of starting treatment (in contrast to only 10% when anti-epileptic drugs alone were used) (114). Early cessation of faciobrachial dystonic seizures with immunotherapy prevented the development of cognitive impairment (all patients were also shown to have IgG4-LGI1 antibodies but those with cognitive impairment had a higher proportion of complement fixing IgG1 antibodies (114, 115).

Lower response rates are seen in encephalitis associated with antibodies to AMPAR and GABA_B_R, which are often associated with cancer. In general patients with antibodies to intracellular antigens (classic paraneoplastic disorders which are predominantly T cell mediated) have a worse prognosis after immunotherapy, compared to those with antibodies to cell surface or synaptic antigens. The frequency of relapses in encephalitides associated with antibodies against NMDAR, AMPAR, LGI1, or CASPR2 can range from 12 to 35%. Long term immunosuppression with Azathioprine or mycophenolate mofetil can be given to prevent relapses although the evidence for benefit is not conclusive.

### Treatment of NMOSD

An acute attack of NMO is treated with high dose corticosteroids (1 gm IV methylprednisolone for 5 days). Most patients should also receive early treatment with 5–7 plasma exchanges. Maintenance therapy is very important as NMOSD is a relapsing disorder. The most commonly used medications for maintenance are oral prednisone along with the steroid sparing drugs azathioprine, mycophenolate mofetil, methotrexate or rituximab. MOG-antibody associated disorders are treated in a similar manner.

New treatment options are becoming available for the treatment of NMOSD. AQP4-IgG1 antibodies are known to trigger the complement cascade, leading to inflammatory CNS damage. Eculizumab a terminal complement (C5) inhibitor was shown in a phase 3 trial to reduce the risk of relapses in NMO by 94% compared to placebo (116). Benefit was maintained long term (median 2.5 years) (117). Another strategy has been to use IL6 receptor blocking monoclonal antibodies such as satralizumab and tocilizumab. Disruption of IL6 signaling might affect disease by reducing AQP4 antibody production, inhibition of pro-inflammatory T cell differentiation and lowering of blood-brain barrier permeability. These agents reduce the relapse rate by about 60%.

### B Cell Depleting Therapy

The most significant advance in the treatment of MS (and to some extent other nervous system autoimmune diseases) has been the advent of highly efficacious monoclonal antibodies which deplete B cells (118, 119). Anti- CD 20 monoclonal antibodies rapidly deplete circulating B lineage cells which express CD20 (a cell surface marker expressed on pre-B, mature and memory B cells but not on stem cells, pro-B cells, plasmablasts or plasma cells). The anti-CD20 antibodies currently being used are Rituximab, a chimeric mouse human monoclonal antibody; Ocrelizumab, a humanized monoclonal antibody, and Ofatumumab a fully human antibody. These therapies lead to rapid and almost complete depletion of circulating B cells (but less so in lymph nodes) for ~1 year after a single course, by antibody dependent and complement dependent cytotoxicity. Since auto-antibody levels are not markedly reduced by Rituximab, the major mechanism of action is believed to be the depletion of naïve and memory B cells, impaired antigen presentation by B cells and decreased release of inflammatory cytokines such as tumor necrosis factorα and lymphotoxin and increase in anti-inflammatory IL-10. Although intrathecal concentration of Rituximab after systemic administration is <0.1% of plasma levels, CSF B cells decrease dramatically after therapy, implying that peripheral depletion of B cells leads to decreased CNS migration. However, CD20^+^B cells still persist in meninges and CNS parenchyma after treatment (120), indicating the possibility of relapses. Also, since profound B cell depletion may lead to increased risk of new infections, reactivation of latent infections such as JC virus, tuberculosis and hepatitis or malignancies, longer term safety issues should be considered.

### Research Into Novel Targeted Therapies for Autoimmune Diseases

Most current treatment choices for autoimmune diseases (Table 4), although effective, involve broad immunosuppression, with its concurrent risk of increased infection or malignancy. An ideal treatment for autoimmune diseases would eliminate auto-reactive immune cells but spare protective immunity.

### Cell Therapy With Immunosuppressive T_reg_ Cells

T_reg_ cells are a small subset, 5–7%, of CD4^+^ cells (CD4^+^, CD25^+^, FOXP3^+^, and CD127_low_), which maintain immune homeostasis by suppressing excessive immune activation and inflammatory damage (121, 122). Several pre-clinical and early clinical trials with polyclonal T_reg_ cells are underway. However, T_reg_ therapy which only targets antigen-specific autoreactive T cells is more potent and has less risk of generalized immunosuppression than using polyclonal T_reg_ cells (123). Strategies to generate antigen-specific T_reg_ cells are: (i) engineering the T cell receptor (TCR) by transducing TCR variable (V) genes from a patient's antigen-specific, auto-reactive T cell clone into T_reg_ cells; (ii) introducing a chimeric antigen receptor (CAR), comprising an extracellular auto-antigenic peptide-MHC molecule linked to a TCR signaling domain into T_reg_ cells. T_reg_ cells engineered in these ways recognize and are activated by MHC-peptide on antigen presenting cells or by pathogenic auto-reactive T cells and mediate localized immunosuppression. For example, myelin basic protein specific T_reg_ cells with an engineered T cell receptor (124) or a CAR (125) have been shown to ameliorate EAE in mice.

### Nanoparticles Coated With Peptide-MHC for Antigen-Specific Immunosuppression

In another successful strategy in animals, nanoparticles coated with peptide fragments from myelin oligodendrocyte protein, bound to MHC Class II proteins were injected into EAE mice (126). On receiving this signal, autoreactive CD4^+^ T cells with receptors for that peptide (in the absence of co-stimulatory signals from antigen presenting cells) expanded and differentiated into an immunosuppressive subset of regulatory T cells, instead of inducing an immune response against the self-protein. This led to further recruitment of auto-reactive B cells as regulatory cells, suppression of antigen presentation and even restoration of strength in paralytic mice (126).

### CAR T Cell Antigen-Specific Immunosuppression

Significant advances in cancer treatment have involved the use of chimeric antigen T cell receptors (CAR-T cells), which are engineered T cell receptors with a single chain extracellular antibody molecule, capable of binding to a specific antigen, and destroying cancer cells expressing that antigen (127). A similar approach for the treatment of autoimmunity involves the generation of engineered T cells which bind specifically and only to auto-reactive B cells and destroys them. This has been validated in a mouse model of an autoimmune disease, pemphigus, caused by B cell production of auto-antibodies to Desmoglein3. T cells with chimeric CAR-T receptors expressing extracellular Desmoglein 3, fused to the zeta chain of the T cell receptor CD3 signaling domain (CD3 ζ), specifically destroyed only anti-Desmoglein3 producing B cells and ameliorated disease in mice (128). Such an approach may be feasible against autantibody-mediated disease where the target antigen is known, such as Aquaporin4 in NMO.

### Positive Allosteric Modulation as a Therapeutic Strategy

NMDAR antibodies binding in patients result in NMDAR loss and hypofunction. Preliminary work suggests that allosteric binding of compounds such as synthetic oxysterols to extracellular regulatory sites of the NMDAR (distinct from the agonist binding and channel permeating sites) can restore NMDAR function in cultured rat hippocampal neurons and prevent the pathogenic effects of NMDAR auto-antibodies in a mouse model, suggesting a potential new strategy to counteract abnormal NMDAR function (129).

Although promising in *in vitro* or animal studies, the ability to translate these innovative approaches to treat human diseases, safely and effectively, is a subject of ongoing research.

## Conclusion

Recent research has outlined a broad range of autoimmune disorders of both the central and peripheral nervous system, which may be triggered by diverse factors in genetically susceptible individuals. Several new disorders such as NMOSD, MOG-antibody associated disease and the various encephalitides caused by antibodies to synaptic receptors in the CNS have been defined, permitting rapid diagnosis and treatment. The antigenic target of this autoimmune attack has been well-defined in many of these disorders, such as in myasthenia gravis, NMDA receptor encephalitis and NMOSD but is uncertain in others such as multiple sclerosis and Guillain Barre syndrome. The autoimmune attack and pathology may be predominantly B cell and auto-antibody mediated, such as in myasthenia gravis and NMOSD or predominantly T cell mediated as in the classic paraneoplastic disorders. Current therapy encompasses eliminating pathogenic auto-antibodies by plasma exchange and broad immunosuppression. Novel research avenues being pursued raise hopes for the possibility of targeted immunotherapy in the future.

## Author Contributions

The entire article, figures and tables have been conceived and written by SB.

## Conflict of Interest

The author declares that the research was conducted in the absence of any commercial or financial relationships that could be construed as a potential conflict of interest.
